# Recent Advances in Computational Mechanics of the Human Knee Joint

**DOI:** 10.1155/2013/718423

**Published:** 2013-02-19

**Authors:** M. Kazemi, Y. Dabiri, L. P. Li

**Affiliations:** Department of Mechanical and Manufacturing Engineering, University of Calgary, 2500 University Drive NW, Calgary, AB, Canada T2N 1N4

## Abstract

Computational mechanics has been advanced in every area of orthopedic biomechanics. The objective of this paper is to provide a general review of the computational models used in the analysis of the mechanical function of the knee joint in different loading and pathological conditions. Major review articles published in related areas are summarized first. The constitutive models for soft tissues of the knee are briefly discussed to facilitate understanding the joint modeling. A detailed review of the tibiofemoral joint models is presented thereafter. The geometry reconstruction procedures as well as some critical issues in finite element modeling are also discussed. Computational modeling can be a reliable and effective method for the study of mechanical behavior of the knee joint, if the model is constructed correctly. Single-phase material models have been used to predict the instantaneous load response for the healthy knees and repaired joints, such as total and partial meniscectomies, ACL and PCL reconstructions, and joint replacements. Recently, poromechanical models accounting for fluid pressurization in soft tissues have been proposed to study the viscoelastic response of the healthy and impaired knee joints. While the constitutive modeling has been considerably advanced at the tissue level, many challenges still exist in applying a good material model to three-dimensional joint simulations. A complete model validation at the joint level seems impossible presently, because only simple data can be obtained experimentally. Therefore, model validation may be concentrated on the constitutive laws using multiple mechanical tests of the tissues. Extensive model verifications at the joint level are still crucial for the accuracy of the modeling.

## 1. Introduction

The human knee is the largest joint in the musculoskeletal system, which supports the body weight and facilitates locomotion. The knee consists of two distinct articulations, the tibiofemoral and the patellofemoral joints [1]. The tibiofemoral joint is one of the most complex articulations of the human body and its main tissues are the femur, tibia, fibula, articular cartilages, menisci, and ligaments. The tibiofemoral joint enables the relative motion of the femur and tibia, which is facilitated through mechanical contacts between the cartilages and menisci [2]. In order to understand common injuries and development of osteoarthritis (OA), extensive experimental and computational studies have been performed on this joint and its individual tissues. Among the computational approaches, the Finite Element Method (FEM) has been widely used to investigate the biomechanics of the knee joint at the cell, tissue, and joint levels.

 The earliest application of FEM in biomechanics goes back to 1972 [3], only over a decade after FEM was introduced as a powerful tool in structural analysis. Since then, FEM has been used in different areas of bioengineering. In 1983, the first review paper on the application of FEM in orthopedic biomechanics was published by Huiskes and Chao [4]. In 1992, Clift reviewed the application of FEM in cartilage biomechanics and investigation of OA [5]. Later, Goldsmith and coauthors reviewed stress analysis of articular cartilage under compressive loading in 1996 [6]. Single-phase and biphasic analytical models of articular cartilage and their FE simulations were discussed in their article along with experimental studies. In a review by Hasler and coauthors, the experimental methods and theoretical models of articular cartilage were discussed, and the material properties for normal, pathologic, and repaired cartilages were summarized [7]. Knecht and coauthors reviewed the studies on the mechanical properties of articular cartilage and provided reference data for the cartilage properties in preosteoarthritis; the data provided can be used in studies of cartilage degeneration and diagnosis of osteoarthritis [8].

 In the last decade, many reviews targeted the constitutive modeling of individual tissues of the knee. Wilson and coauthors reviewed the computational and analytical models of articular cartilage proposed for the study of mechanical behavior and damage mechanisms. The models they reviewed included swelling and chemical expansion [9]. Taylor and Miller summarized the macroscopic and microstructural constitutive models of cartilaginous tissues [10]. At the macroscopic level, as in single-phase and biphasic models, the bulk mechanical behavior of the cartilage was discussed with no consideration of the microstructural components of the tissue (such as collagen fibrils). The microstructural models include the fibril-reinforced and swelling models, as discussed in the review. van Donkelaar and Schulz [11] discussed the patents for mechanical stimulation of cartilage transplants and chondrocyte-loaded scaffolds using bioreactors. Although the paper does not discuss the constitutive modeling, it provides useful information for FE modeling of tissue-engineered cartilages. Woo and coauthors reviewed the mathematical models of ligament with special focus on viscoelastic models. In particular, they compared the theory of quasi-linear viscoelasticity (QLV) with the single integral finite strain model [12]. Weiss and coauthors evaluated the computational models of ligament in one-dimensional and three-dimensional scales with focus on the relationship of microstructures and the continuum mechanical behavior [13, 14]. Beside the numerical aspects, the experimental studies to obtain the material properties of ligaments were also discussed in their work [13]. Provenzano and coauthors reexamined the nonlinear viscoelastic models of ligaments based on the existing experimental data and evaluated their ability to predict the dependency on strain amplitude and frequency [15].

Despite extensive analytical and computational studies on the human knee joint, few review papers in this area are available in the literature. Hefzy et al. reviewed the analytical models of knee joint used to describe the knee kinematics and kinetics [16] and later updated the review [17]. Those analytical models use rigid body mechanics and usually ignore the deformation of tissues, such as cartilage and menisci. Peña and coauthors reviewed the computational models of human knee and temporomandibular joints with major focus on the visco-/hyperelastic constitutive behaviors of soft tissues, including muscles, ligaments, tendons, and articular cartilage as single-phase materials [18]. Elias and Cosgarea reviewed different computational aspects of the patellofemoral joint including modeling techniques, for example, patient-specific modeling, and clinical applications [19]. Mackerle published a bibliography spanning 1998–2005 in modeling and simulations in orthopedics. The bibliography provides an extensive list of publications in different areas of computational biomechanics including knee and hip joints [20].

 The objective of this paper is to provide a general review of the computational models of the knee joint proposed for different biomedical/clinical applications. For brevity, the focus of our paper will be on the FE models of the tibiofemoral joints, with some examples of the patellofemoral joints. The constitutive models for soft tissues of the knee are briefly discussed. The geometry reconstruction procedures as well as some issues in finite element modeling are also covered. A comprehensive review of published joint models is presented thereafter. Representative articles on different aspects of the knee biomechanics, including general contact behaviors, ACL and PCL reconstruction, meniscectomy, knee replacement, and experimental validation, are reviewed. Finally, the remaining challenges and possible future directions in this area are discussed.

## 2. Constitutive Modeling of the Tissues

Several constitutive models have been developed to simulate the mechanical response of individual tissues of the knee in 1D or 2D geometries. These models may provide stress-strain relationships for 3D studies of the knee. We do not intend to review the constitutive models of the tissues but provide a brief summary of constitutive description to facilitate our review on the computational studies of the knee joint.

 Among all soft tissues of the knee, articular cartilage has been of great interest due to significant impact of OA on the quality of life. Cartilage is composed of a porous matrix saturated with water. About 68%–85% of the weight of cartilage is water [21]. The porous matrix is composed of chondrocytes, collagen fibers (mainly type II), and negatively charged proteoglycans. Collagen and proteoglycans form about 50–70% and 30–35% of the matrix dry weight, respectively. The fiber orientation in mature cartilage varies with depth: parallel to the articular surface in the superficial zone, random in the middle zone, and perpendicular to the bone interface in the deep zone [7, 22].

 In the past four decades, extensive studies have been performed to understand the sophisticated behavior of articular cartilage and improve constitutive modeling. The early constitutive models of articular cartilage were single-phase, that is, only the solid phase of the tissue was considered [23–28]. These models have limited capabilities in describing the time-dependent response of cartilage, which is mainly due to the interstitial fluid flow when the tissue is in compression. Viscoelasticity was considered in some of these models to describe the time-dependent response of cartilage [24, 25, 27]. However, single-phase viscoelastic models do not describe the fluid flow in the tissue. The effect of fluid pressure on the tissue stiffness is included in the overall Young's modulus, often called the effective modulus, which is naturally higher than that for the drained tissue [29, 30]. Obtaining the effective modulus is often challenging because the pressure is time and strain-rate dependent [31–33].

 Poroelastic and biphasic models that considered both solid and fluid phases were the second generation of constitutive models proposed to account for the effects of fluid pressurization. The poroelastic models were based on the Biot theory of soil consolidation [34, 35] and in biomechanics were first used to simulate the skull and other bony structures [36–38]. In 1980, the linear biphasic theory was proposed for articular cartilage [39] and then further developed to include variable permeability [40] and large deformation [41, 42]. Although the field equations in the linear biphasic theory are different from the poroelastic equations, it was proved that both linear theories are equivalent for the case of inviscid fluids [43]. However, some inconsistencies were reported in correlating the material properties defined in these two theories [44]. Both poroelastic and biphasic models had limited capabilities in describing the short-term, time-dependent response when the compressive strain-rate was high. One of the reasons is because the fluid pressure was relatively high as compared to the compressive stress in the tissue matrix [45, 46]. Testing of articular cartilage showed that the effective modulus at fast compression could be one order of magnitude higher than that at slow compression [31].

 The fibril-reinforced models were proposed to account for high fluid pressurization in the tissue [47, 48] and may be considered as the third generation of the constitutive models for cartilage. In contrast to a nonfibril-reinforced poroelastic/biphasic model, a fibril-reinforced model could reasonably predict the stresses in the cartilage under fast compressions [33]. The fibrillar nonlinearity was an important factor for modeling high strain-rate compression of articular cartilage; a linear fibril-reinforced model is not sufficient for the description of the load response of cartilage at fast compression.

 The triphasic models were proposed to account for the ion phase in the proteoglycan matrix as the third phase in addition to fluid and solid phases [49]. The overall negative charges of the proteoglycans contribute to cartilage swelling and enhance the tissue stiffness [49, 50]. The triphasic theory was later extended to account for multielectrolytes and polyvalent ions by Gu and coworkers [51]. Although triphasic models provide more specific data about cartilage properties, biphasic and fibril-reinforced models are still widely used in the literature for cartilage modeling.

 Ligaments restrain joint motion to stabilize the joint. These tissues consist of a proteoglycan matrix reinforced by collagen fibers (mainly type I) and elastin. Approximately, 60–70% of the ligament weight is water [13]. The collagen bundles are mainly aligned in the longitudinal direction to provide high stiffness for the ligaments. The elastin content is normally about 1% of total ligament weight and provides the elastic recovery of the tissue [52, 53].

 Extensive computational models have been proposed for ligaments and tendons. Since the ligaments mechanical response is dominated by the collagen fibers, the majority of proposed models focused on the collagen constitutive behavior to predict the ligament response. Fung proposed a one-dimensional constitutive model based on an exponential stress-strain relationship accounting for nonlinear behavior of ligament under finite deformations [54]. Hildebrandt and coauthors later extended Fung's model to biaxial and three-dimensional cases [55]. Some other models were proposed assuming strain rate independence and negligible hysteresis effect; that is, the time-dependent response was neglected and elasticity was assumed. In these one-dimensional studies, bundles of linear elastic elements were used to model ligaments. To capture the nonlinear behavior of the tissue, individual linear elastic fibers in a ligament were assumed slack when the ligament was not externally loaded and were recruited gradually in resisting increased tension [56–61].

 Strain energy and hyperelasticity have been used in studies of ligaments [62–68]. Lanir proposed a strain-energy-based method to describe the three-dimensional behavior of the ligament [62]. The matrix response was simplified as hydrostatic pressure, and the majority of the total strain energy was resulted from the stretch in collagen fibers. Weiss and coauthors proposed hyperelastic continuum models of ligaments based on the incompressibility assumption [64, 67]. In their modeling, collagen fibers, ground substance matrix, and the fiber-matrix interaction contributed to the tissue response. Incompressibility was enforced in their models based on the assumption that fluid is trapped in the tissue during loading, and therefore no fluid exudation occurs.

 Due to intrinsic viscoelasticity of collagen fibers and fluid exudation from the solid matrix, the ligament response is time-dependent. Many studies have considered the viscoelasticity of ligaments using spring-dashpot modeling [57, 58, 69, 70], assuming fibers matrix and fiber-fiber friction [71] or using continuum mechanics approach [72–75]. Among all proposed models, the quasi-linear viscoelastic theory (QLV) developed by Fung [54, 72, 76] has been commonly used in computational studies, probably because of its simplicity. The fluid flow was incorporated in a few studies using theory of poroelasticity [77, 78]. The fluid flow and its relevant tissue response under uniaxial tensile, stress relaxation, and cyclic loadings have been studied using these models.

Ligaments have been commonly modeled as spring elements in the 3D models of the knee joint (Table 1). Nonlinear material behavior (normally quadratic stress-strain relationship) is often used for the toe region up to ~6% tensile strain, which is twice of the so-called nonlinear spring parameter [79–81]. The stress-strain relationship for strains greater than 6% is considered linear. The tensile stiffness of the spring elements can be determined accordingly, provided that the ligament geometry is known. The compressive stiffness is taken to be zero, because the ligament does not support load when it is slack. Some level of prestrain exists in ligaments before the joint is subjected to external loads (ACL, MCL, and LCL are in pretension and PCL in precompression) [79, 82, 83], which are often incorporated into the material model of the ligaments. In addition to spring elements, some studies considered 3D representation of the ligaments in which these tissues were modeled as hyperelastic [84, 85] or fibril-reinforced poromechanical [86–88].

 The menisci are of crescent-like shape and located between the femoral and tibial cartilages, attaching to the tibia via ligamentous tissues called menisci horns [1]. The wedge-shape cross section of the menisci provides the joint congruency and minimizes the direct contact between the femoral and tibial cartilages [89]. Menisci support and redistribute a portion of the joint load, improve joint stability, and facilitate lubrication [90–92]. Some studies also suggest the menisci act as a shock absorber [91, 93, 94], while others do not support this hypothesis [95]. It is estimated that the menisci are subjected to 45%–75% of the joint load, depending on the knee loading and health state of the tissue [2]. The major constituents of the meniscus are fluid, proteoglycan matrix, and collagen fiber (mostly type I) [21]. Water is the most abundant constituent and is about 60–70% of the tissue weight [21]. Collagen fibers weigh about 15–25% and proteoglycans in the range of 1-2% [21]. The fibers in the menisci are mostly oriented in the circumferential direction [96], which redistribute the load in terms of hoop stresses [92, 97, 98]. 

 The mechanical response of meniscus is time-dependent due to fluid flow and intrinsic viscoelasticity of the collagen fibers. However, in the early finite element models of the menisci, these tissues were represented as axisymmetric with single-phase linear elastic properties in contact with deformable bones [99]. In an improved axisymmetric model, nonlinear material behavior in circumferential direction was considered [100]. Transversely isotropic behavior and axisymmetry were considered in some later FE models of the menisci [101, 102]. In a parametric axisymmetric FE study, isotropic, orthotropic fiber-reinforced, and poroelastic models were compared [103]. The fiber reinforcement was concluded in this study to be an essential part of the menisci modeling. Spilker and coauthors developed a biphasic model of the menisci with transversely isotropic behavior for the solid phase of the tissue. Linear biphasic theory was used in their study [104]. Wilson and coauthors used the consolidation theory in ABAQUS for the biphasic modeling of the menisci with axisymmetric representation. Transversely isotropic properties were also used in their study [105]. Hyperelastic material properties have been quantified for the menisci horns in a study by Abraham and coauthors [106]. In 3D models of the knee joint, menisci are generally modeled as single-phase materials represented by spring elements [81, 107, 108], isotropic solid [84, 109], transversely isotropic solid [110–112], or fiber-reinforced materials [80, 113–116]. Recently, fibril-reinforced poromechanical models of the menisci have been incorporated in 3D modeling of the knee joint [87, 88]. Menisci horns are commonly modeled as spring elements [112] or the menisci are fixed at the insertion sites [87]. Table 1 includes a full list of different material models for the different tissues of the knee joint. 

## 3. Computational Models of the Knee Joint

Knee joint models can be classified into analytical and computational. Analytical models were used to describe the knee kinematics and extract information about the joint kinetics. The deformation of tissues except for ligaments is normally ignored in these models and only rigid body motions are studied. This methodology is often called inverse dynamics (rigid-body dynamics) and can be referred as analytical since only minor numerical work is involved for the solutions (we refer to it as analytical in this paper, although some numerical work is involved). Analytical models with different degrees of accuracy have been published in the literature. These models were used to describe the joint motion and kinematics in 2D/3D and to predict the loads in muscles, tendons, and ligaments [79, 107, 117–128]. In some of these models (mostly 2D), simple contact algorithms such as Hertz contact approach were used to describe the tissue interactions [123, 129, 130]. Some analytical models considered geometrical nonlinearities [120, 130] and often included the inertial effects of bones [131, 132]. In some recent studies, rigid-body musculoskeletal models were combined with the FE approach to investigate the contact mechanics of the knee and the role of menisci in the joint functioning [133, 134].

 Validation is a necessary step in the model development. Established data may help researchers to validate their kinematic and rigid body models. The Grand Knee Challenge project provides a database where *in vivo* knee data such as tibia contact force, muscle forces, and ground reactions are available [135]. Although analytical models offered robust approaches to determine knee kinematics, they had limited capacities to describe the stress/strain patterns of cartilages, menisci, and ligaments in 3D configurations. Moreover, the nonlinear, anisotropic, and time-dependent response of the soft tissues could not be captured using these models. Furthermore, analytical models were not suitable for the simulation of the highly nonlinear mechanical contact between articulating surfaces undergoing large deformations. A more comprehensive review of the analytical models can be found in the reviews by Hefzy et al. [16, 17]. The present review is focused on computational joint models. 

### 3.1. Geometry and Mesh Generation of the Knee

The geometry of the knee joint is normally reconstructed from a stack of images obtained from Magnetic Resonance Imaging (MRI), Computed Tomography (CT), or Micro-CT of the joint. The MRI images are usually preferred for the reconstruction of soft tissues, whereas CT images are more accurate for hard tissues (bones). Image processing software packages, such as Mimics (Materialise, Leuven, Belgium) and Simpleware (Exeter, UK), and geometric modeling packages, such as Rhinoceros 3D (Seattle, WA, USA), can be used to reconstruct the 3D geometry from 2D images. The essential process in geometry reconstruction is to precisely select the boundaries of the tissues from the images. This process is called segmentation and can be performed automatically or manually [14]. After the initial geometry is extracted from the images, based on our experiences, some extra editing is normally required to improve model accuracy and smooth the surfaces. This is usually done by eliminating the artifacts, such as redundant edges/vertices, small gaps, and sharp edges, that may result in impossible meshing or unnecessary dense meshing. If necessary, some software packages such as Geomagic (Morrisville, NC, USA) can be used to improve the quality of the surface geometry.

**Figure 1:**
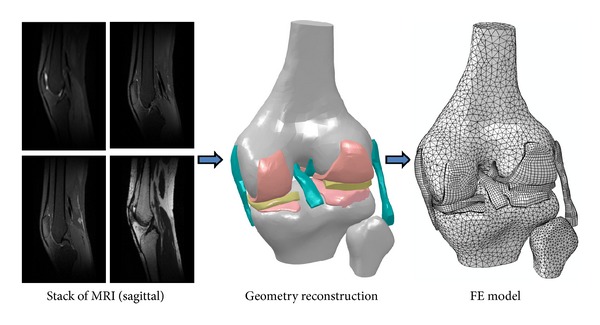
A schematic representation of geometry reconstruction from MRI data and FE mesh generation.

The FE mesh can be generated using the built-in functions of the image processing software. Alternatively, the meshing process can be performed in FE programs, such as ABAQUS (Simulia, Providence, USA), or in specialized meshing programs, such as HyperMesh (Altair, Troy, MI, USA). The choice between meshing tools of an image processing software and a third party meshing program is mainly based on the required mesh type. The imaging software we have used, such as Mimics, provides limited control over meshing. If one needs pure hexahedral elements, for example, imaging software may not be able to perform the meshing [14, 136]. If no specific mesh type (e.g., tetrahedral versus hexahedral) is required, it is more convenient to use the built-in meshing tools of the image processing programs to generate an automatic mesh. It normally yields triangular/tetrahedral elements or a combination of tetrahedral and hexahedral elements. Using this approach, the mesh information (nodal coordinates and element numbers) can be normally exported to an FE software to perform finite element analysis. However, since the exported mesh (usually called orphan mesh) does not include all geometric information of the reconstructed knee, any major changes in the mesh, or mesh regeneration, can be only performed in the image processing software. Therefore, if a structured mesh (mapped mesh) of pure hexahedral elements is required, or the unmeshed tissue geometry (in addition to the FE mesh) is needed during the FE simulations, the reconstructed geometry should be exported into the FE software or a third-party meshing program to generate the mesh.  Figure 1 illustrates a schematic of knee geometry reconstruction and mesh generation from MRI data.

### 3.2. Implementation of Tissue Models

Due to computational costs and convergence difficulties associated with 3D modeling, simpler constitutive laws have been commonly used in whole joint simulations as compared to the studies on the mechanics of a single tissue (see Section 2). For instance, single-phase material model has been widely used for cartilages and menisci in knee joint modeling [80, 81, 108–111, 113, 137]. Fluid pressurization has not been incorporated in 3D joint modeling until recently [86–88, 112, 138–140]. In a majority of joint models, bones were considered as rigid because of their higher stiffness compared to the cartilaginous tissues. Articular cartilages were commonly modeled as single-phase, linear elastic, homogenous, and isotropic materials with constant stiffness [80, 81, 84, 113, 141]. Due to the high viscoelastic time constant of cartilage [28] (~1500 s), there is no time for fluid flow at the instant of loading, and thus, the tissue may be considered as a single-phase material with a large equivalent elastic modulus for the short-term response. However, if the loading is not fast or if the time-dependent response of the knee is sought, the single-phase aclssumption is not satisfactory [32, 87]. Furthermore, a compressible material model may not be used to predict the instantaneous response of the tissue [32, 87]. Menisci were commonly considered as linear elastic, isotropic [84, 109, 142], transversely isotropic [110], or linear elastic solid with fibril reinforcement [80, 113, 137]. Ligaments were usually modeled by 1D spring/bar elements [80, 81, 110, 113, 137, 143], and in some cases, 3D and hyperelastic elements [84, 109, 142]. Table 1 summaries different constitutive models of knee tissues used in joint mechanical simulations.

### 3.3. Finite Element Model Developments

One of the first FE models of the knee joint was proposed by Chand and coauthors in 1976 [144]. The contact stress between femur and tibia, in the absence of soft tissues, was investigated. A 2D model of the knee generated from X-rays of a live subject was used in their simulations. The FE software NASTRAN (MSC Software Corporation, Santa Ana, CA, USA) was used to obtain the force-deformation relations, and a numerical approach based on Wolfe's algorithm was developed to solve the nonlinear equations. Brown and coauthors used a simplified axisymmetric model of articular cartilage and subchondral bone to study juxta articular stress changes due to localized subchondral stiffening [145]. Huber-Betzer and coauthors developed a plane-strain FE model of the knee including bones and cartilages using ABAQUS and FEAP (University of California, Berkeley, USA) programs [146]. The model was used to study the contact stress distribution associated with joint incongruity. The effects of joint surface curvature, cartilage stiffness, and thickness were investigated in their study.

Heegaard and coauthors developed a FE model of the human patellofemoral joint including bones and articular cartilage and calculated the contact stresses and ligament/tendon forces during passive knee flexion. The patella geometry was reconstructed using CT images in the sagittal plane [152]. Besier and coauthors developed a 3D FE model of the patellofemoral joint using MRI data. The model included bones and cartilage and an estimate of muscle forces. The stresses and strains in the cartilage were calculated and some of the results such as contact area obtained from simulations were compared with the experimental data [153]. They further examined the effect of internal-external knee rotation on the mechanics of patellofemoral joint, using FE models reconstructed from MRI of 8 male and 8 female subjects. It was found that an external femoral rotation of 15° increased patellar peak shear stress by 10% in more than 75% of the subjects. The stress in cartilage was reported to change considerably from subject to subject, which could have clinical implications [154]. Farrokhi and coauthors predicted higher hydrostatic and octahedral shear stress in the patellofemoral joint for the subjects with patellofemoral pain, as compared to the pain-free subjects, supporting stress-reducing treatment strategies [155]. Fitzpatrick and coauthors compared FE and rigid-body analyses of the patellofemoral joints of eight subjects. Parameters of the rigid contact were based on elastic foundation theory (e.g., [79]). The same geometric properties, for example, cartilage thickness, were used in both rigid-body and FE analyses. Obtained results indicated that the rigid body analysis yields reasonable and yet efficient solutions in terms of accuracy and computational time [156].

 Bendjaballah and coauthors investigated the biomechanics of the tibiofemoral joint using a 3D FE model of the knee including soft and hard tissues undergoing large deformations. CT images were used to reconstruct the knee geometry. An in-house nonlinear FE program was used to perform the simulations. The contact stresses of the healthy and meniscectomy knee joints were studied under compressive loading [80]. Further studies were performed on the knee contact mechanics under drawer (anterior posterior) forces as well as varus-valgus and internal-external rotations [114–116]. Périé and Hobatho investigated the contact areas/pressures of the knee joint in full extension using ABAQUS. It was found that the predicted hydrostatic pressures were higher in the medial compartment of the joint [157]. (Note: the hydrostatic pressure here is not the pore fluid pressure. It is the average of the three normal stress components).

Moglo and Shirazi-Adl studied the screw-home mechanism, which is the rotation between the tibia and femur during knee passive extension/flexion: during knee flexion, the tibia undergoes internal rotation, whereas during knee extension tibia undergoes external rotation. They also investigated the coupling between the cruciate ligament forces under flexion extension. It was found that ACL transection and changes in initial strains in ACL affect the screw-home mechanism. Moreover, a significant coupling was observed between the ACL and PCL forces in knee flexion [158]. An increase in the initial strains (or pre-tensions) in the ACL or PCL resulted in an increase in the forces of both ligaments. Similarly, when either the ACL or PCL was cut, the forces in both ligaments were diminished. Mesfar and Shirazi-Adl further considered both tibiofemoral and patellofemoral joints. The knee response in flexion under quadriceps forces was investigated in their study using anatomically accurate models of the knee [159]. 

 The effects of bone deformations and boundary conditions on the contact mechanics of the knee were also investigated. Frictionless finite sliding contact was assumed between the articulating surfaces. It was found that rigid body assumption for bones changed the contact stresses by less than 2%, whereas fixing the rotational boundary conditions other than flexion extension had significant impact on the results [110]. Haut Donahue and coauthors also investigated the impact of meniscal material properties on the predicted contact stresses. They reported a considerable sensitivity of contact pressures to the circumferential stiffness of the menisci [97].

 An explicit dynamic FE method was used to study gait biomechanics of the knee [160]. The knee flexion up to 25 degrees was simulated in the study. An FE model of the lower limb was developed to investigate the *in vivo* knee response under impact loading. An explicit FE was employed with consideration of large deformations [150].

**Figure 2:**
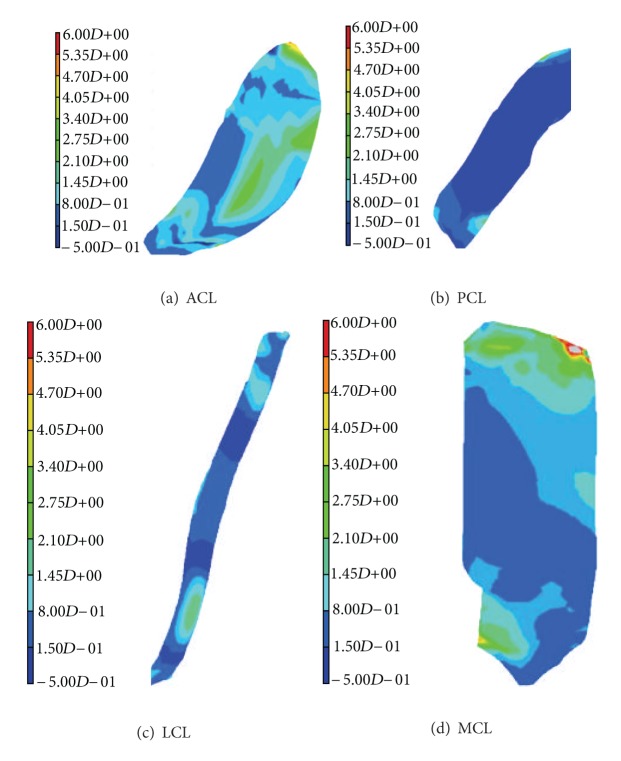
FE computed maximal principal stress (MPa) in ligaments: ACL (a), PCL (b), LCL (c), and MCL (d). The knee was subjected to a compressive load of 1150 N and a valgus compression of 10 Nm (reproduced from [84] Elsevier license permission 3020920850913).

 Shirazi and coauthors implemented the depth-dependent fiber reinforcement in articular cartilages in their knee joint model. The role of collagen network was investigated in their study under compressive forces. It was found that deep vertical fibrils played an important role in the load support mechanism of cartilage *in situ* [113]. In all the previously mentioned studies, spring elements were used for ligaments. Peña and coauthors developed a knee model including more realistic geometries of all ligaments. Transversely isotropic, hyperelastic properties were considered for ligaments, and their roles in knee stability and load transmission were investigated. Eight node hexahedral elements were used to mesh the ligaments (Figure 2) [84]. Dhaher and coauthors investigated the effects of connective tissue material uncertainties on the joint biomechanics. Probability density functions with Gaussian distribution were used to alter the material properties. Based on a multifactorial sensitivity analysis, they reported a significant effect of ACL properties on the knee biomechanics during knee flexion [85].

 Some researchers predicted the mechanical response of chondrocytes based on multiscale modeling of the knee joint [161, 162]. Implementing a multiscale framework, Sibole and Erdemir [161] determined the cellular microscale parameters using the results of a macroscale FE model of the knee. Deformation gradients computed at the joint-level were used to prescribe the boundary conditions of two cell-level models, which included one and eleven cells, respectively. The multiscale modeling was believed to be capable of predicting the cellular deformation metrics such as change in cell's aspect ratio and maximum shear strain resulting from the joint loading [161].

### 3.4. Poromechanical Models

Although fluid flow and pressurization play an essential role in the mechanical functions of articular cartilage and meniscus, it has not been considered in the anatomically accurate knee modeling until recently [138]. In previous studies, only the elastic behavior of the knee was investigated, including the static equilibrium response as well as the instantaneous response of the joint at which no fluid flow occurs. In most of the studies, a large effective modulus and a Poisson's ratio close to half were used to approximate the incompressible behavior of the knee at instantaneous compression. However, only if the fluid pressurization is implemented, the time-dependent response of the knee, and in particular, stress relaxation and creep phenomena may be predicted. For example, a prolonged standing can be modeled as a creep problem.

 Before fluid pressurization was implemented into any anatomically accurate knee models, it had been considered in geometrically simplified contact models. Ateshian and coauthors developed a finite sliding, frictionless contact algorithm for porous media that could be used to simulate 3D cartilage layers in contact [163]. Wilson and coauthors used an axisymmetric model of the cartilages and menisci for the study of meniscectomy [105]. Adeeb and coauthors investigated the effect of joint congruency on the load bearing mechanism of the knee using axisymmetric cartilaginous tissue layers. They concluded that the existing natural incongruence of the joint had a significant impact on the stress and fluid pressure distributions. Their study suggested an important role of the meniscus in the load bearing mechanism of the knee joint [164].

 One of the first 3D computer models of the human joints that included fluid flow was constructed with ABAQUS by del Palomar and Doblaré for the investigation of the internal derangement of the temporomandibular joint [140]. Gu and Li developed the first anatomically accurate tibiofemoral joint model accounting for fluid pressurization and fibril-reinforcement in cartilages and menisci [138]. They also considered the fiber orientations in the femoral cartilage and menisci. Their results indicated a substantial role of fluid pressurization in the mechanical functions of the knee. In a further study, Li and Gu compared the instantaneous response of the knee predicted by a fibril-reinforced model with that obtained from a single-phase compressible elastic model. Substantial differences were found between the two models [32]. In particular, choosing a constant effective modulus in the elastic model might not be satisfactory for different magnitudes of compression.

**Figure 3:**
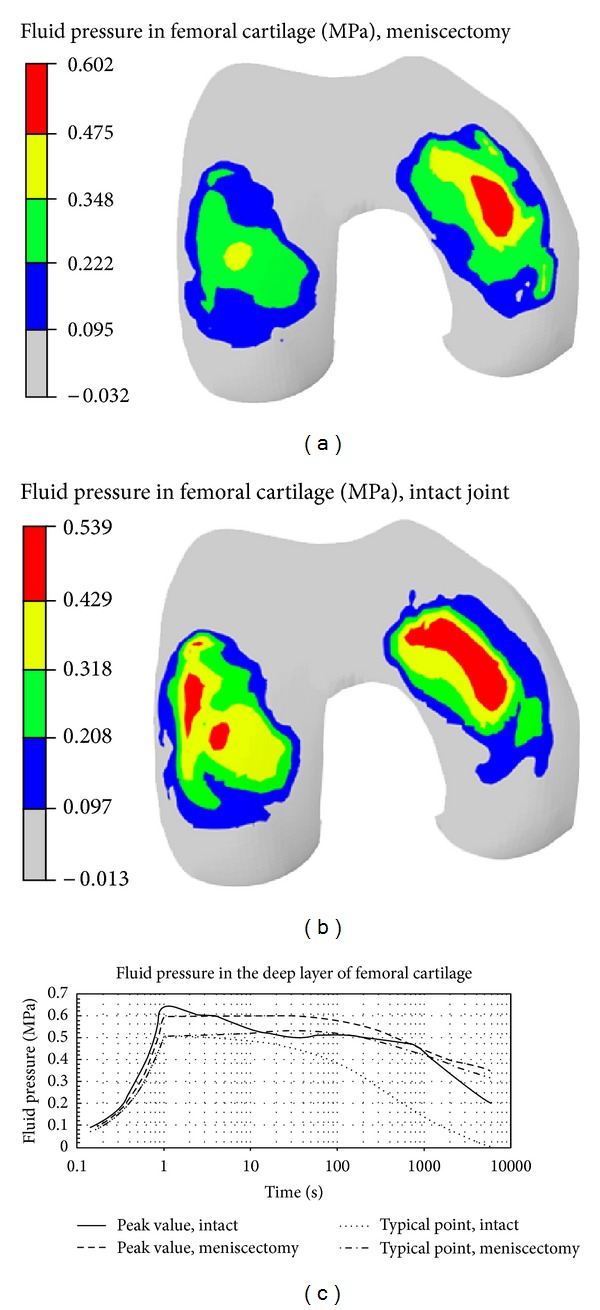
Fluid pressure in femoral cartilage of the meniscectomized (a) and intact (b) knees. The change in fluid pressure of the intact and meniscectomized joints, with respect to time, is shown in (c) (reproduced from [87]; Elsevier license permission 2927920112090).

 Kazemi and coauthors investigated the creep behavior of the intact and total meniscectomized knees under compression (Figure 3). They reported substantially different creep behaviors and contact mechanics of the healthy and meniscectomized knees [87]. In a further study, they investigated the impact of the location and size of partial meniscectomies on fluid pressurization of articular cartilage under stress relaxation and creep loading. They observed a significant increase in fluid pressure and its gradient as well as substantial alterations in pressure distributions after partial meniscectomy [86, 88]. Mononen and coauthors used an axisymmetric, fibril-reinforced model of the cartilages and menisci to study the impact of OA on the stresses in the collagen network of cartilage. They predicted decreased stresses in the superficial zone of cartilage with osteoarthritis. They speculated that collagen fibrillation increased from the superficial zone to the deep zone during progression of osteoarthritis [139]. They also used a fibril-reinforced model of cartilages in contact with single-phase menisci to study the effect of superficial collagen patterns with a 3D knee model. They suggested a significant role of split-line patterns on the strain and stress patterns but a minimal role on fluid and contact pressures [112].

## 4. Verification of the Numerical Modeling

Verification examines the accurate implementation of the mathematical equations, numerical procedures, and computer codes. A verified computational model is an accurate representation of the corresponding methodology. However, a successful thorough verification does not mean that the computational model accurately mimics the physics of the problem. Validation is to examine whether the model reproduces the real-world problem and thus must be done through measurement (see Section 5 for validation). For general information about verification and validation procedures, the readers are referred to the guide for verification and validation published by the American Society of Mechanical Engineers [165] and other articles [166–169]. Some specific issues of model verification are presented here.

 The verification of anatomically accurate knee joint models includes a few aspects of the model construction, including image segmentation, geometry reconstruction, finite element meshing, initial and boundary conditions, contact definition, and solution procedure. Most of the computational knee models are constructed using commercial FE software such as ABAQUS. The numerical procedure of commercial software packages has been to some extent tested and verified by the developing teams and independent researchers [14, 170–172]. While the solution procedure of the commercial software packages is generally verified, especial attention is required on other aspects of FE modeling such as meshing, material parameters, and boundary conditions. Moreover, if a custom code is used for the computational modeling and solution, a comprehensive verification is required regarding the numerical implementation and solution procedure.

**Figure 4:**
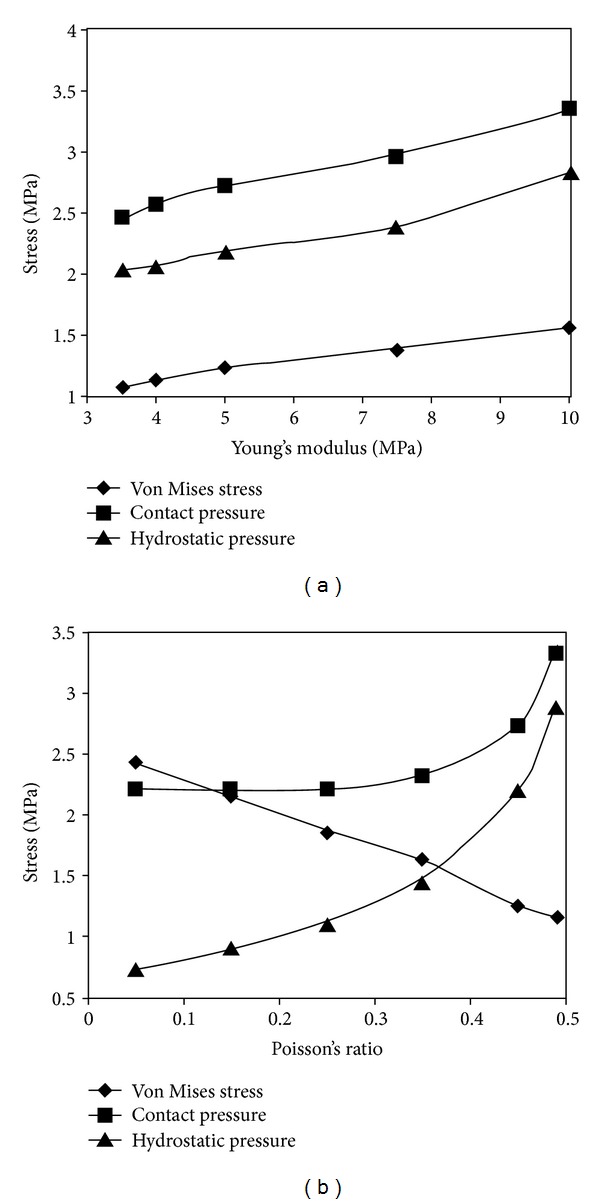
Variations in contact pressure, von Mises stress, and hydrostatic pressure with material properties (reproduced from [143]; ASME permission 341–346; royalty paid 1074929380).

 The sensitivity of FE model to the reconstruction procedure was investigated by generating five knee models of the same joint using the same set of MR images. Each model was independently reconstructed by a different researcher [143]. It was found that the deviations of cartilage thickness in five models resulted in approximately 10 percent difference in peak contact pressure. The sensitivity of material properties was also examined in the study. It was observed that the results were more sensitive to Poisson's ratio than Young's modulus. The von Mises stress decreased and the hydrostatic pressure increased with increased Poison's ratio of cartilage (Figure 4). Large deformations were considered. An optimization approach was developed to determine the equivalent stiffness of the springs that were used to model ligaments and menisci. Articular cartilage was considered as a single-phase material [143].

 Two knee models were reconstructed from the CT and MR images of the same cadaveric knee joint using Analyze II (Mayo Biodynamics Research Unit). They were compared with measurements obtained from implanted reference markers using a 3D digitizer machine. Results showed comparable accuracy of the reconstructions from the MR and CT images [173].

 With a 3D FE model of the knee joint, Donahue and coauthors examined the effect of rotation constraint and bone rigidity. MSC/Patran (MacNeal-Schwendler Corp., Santa Ana, CA, USA) and TrueGrid (XYZ Scientific Applications Inc., Livermore, CA) were employed to reconstruct the geometry using data from CT and 3D coordinate digitizing system. ABAQUS was used for the FE analysis. The FE model was verified against the mesh size using average element sizes ranging from 5 by 5 mm to 1 by 1 mm. The average element size of 2 by 2 mm yielded a convergent result [110]. Hao and coauthors examined the sensitivity of their knee model to mesh sizes of 3.0 mm, 2.5 mm, and 2.0 mm. They reported a maximum of 3% change in the contact pressure when the mesh was refined from 2.5 to 2.0 mm [160]. Peña and coauthors investigated the convergence of their knee model by double increasing the mesh density. They found a maximum of 4% change in peak contact stresses with the double-dense mesh compared to the original mesh [84].

 It was found from a hip FE modeling that errors in cartilage shear modulus, bulk modulus, and thickness had higher influence on the peak pressures, as compared to the average contact pressure and area (±25% compared to ±10%). This study also indicated possible errors of the rigid bone assumption for simulating certain activities, such as stair descending. The labrum was not included in the modeling [174].

## 5. Validation of the Numerical Modeling

**Figure 5:**
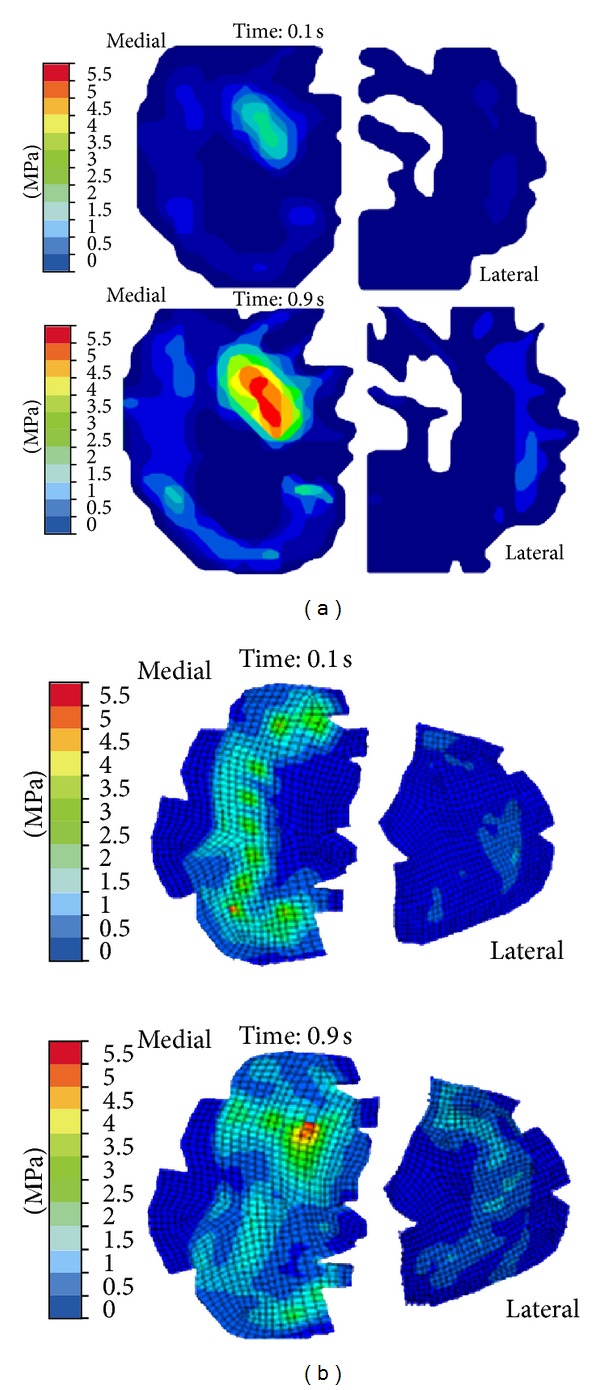
Contact pressures at the tibia plateau measured by Tekscan K-scan sensor (a) and computed by finite element analysis (b) (reproduced from [147] Elsevier license permission 3020921021572).

The experimental validation of computational knee models is challenging due to difficulties in measurements [175–178]. For example, specialized Fujifilm and Tekscan pressure sensors may be used to measure the contact pressure in the joint (Figure 5). However, the insertion of the film or sensor somehow alters the contact in the joint due to the thickness and stiffness of the film or sensor. Therefore, the data measured are more or less compromised. It is worth mentioning that a complete validation of a computational model requires multiple data at different levels. For instance, one may validate the global kinematics/kinetics, such as femoral displacement/forces, against experimental data. However, this does not necessarily mean that the stresses and strains can be accurately predicted by the model. A more reliable method is a simultaneous validation of the stresses and joint force, for example. We herein first summarize some experimental techniques that may be or have been used to validate the numerical models and then review some experimental validations of joint modeling. Note that some validations are presented in other sections when the relevant models are reviewed.

 The casting method has been used to measure contact areas in the joint. This method is based on the formed pattern of a material such as silicone rubber or polymethylmethacrylate cast around the joint contact. Based on this method, Walker and Hajek determined contact areas and locations of cadaver knee joints under a force applied along tibia, for different flexion angles, and found larger contact areas in the medial condyle. Contact areas were decreased as the knee flexion angle increased [179]. Fukubayashi and Kurosawa added Prescale sensors (Fuji Film Co., Ltd., Tokyo) to the casting method to measure the contact pressure and area of the tibiofemoral joint in full extension. They found that the removal of the menisci from a healthy knee considerably increased the contact pressure and decreased the contact area in the joint. In contrast, the removal of menisci from a osteoarthritic knee had less impact on the change of contact pressure and area [180]. Further experiments showed the average contact stress to increase by 2-3 times when the menisci were removed [91].

 The contact locations in cadaver knees during high flexion were mapped based on the fiducial points on each bone recorded for the position, by the use of reconstructed bone geometries [181]. Rhinoceros and Rapidform (Inus Technology Inc., Seoul, Republic of Korea) software packages were used to reconstruct the geometry of each bone from digitized surface data. The contact areas for a given knee flexion were derived from the bone surface geometries and the bone positions corresponding to that knee flexion [181].

 Brown and Shaw measured the contact stress in cadaveric knee joints at different flexion angles using arrays of piezoresistive transducers. They studied healthy knees as well as medial and dual meniscectomy cases. Results showed that in normal knees the medial femoral condyle supports higher load compared to the lateral condyle, but after removal of the medial meniscus the load was transferred slightly to the lateral condyle. It was found that in the flexion range of 0 to 30 degrees, the size of contact area and the magnitude of contact stresses were not changed significantly although the contact location changed during the flexion. Furthermore, compared to previous experimental studies, they suggested a moderate decrease in contact areas and increase in contact pressures following meniscectomy [182].

 There is an increasing trend to use imaging technology to determine tissue deformation under external loading. Herberhold and coauthors measured the deformation of femoropatellar articular cartilage from cadaver specimens using MRI [183]. Segmentation, reconstruction, and image analyses were performed using an in-house code. Fluid flux and deformations of femoral and patellar cartilages under 150% of body weight were obtained [183]. Liu and coauthors used MRI with fluoroscopic system and Rhinoceros imaging software to determine the knee kinematics during stance phase of gait. They reported higher contact deformation in the thicker regions of cartilage and larger contact area in the medial compartment than the lateral compartment [184]. Li and coauthors measured contact locations in the knee joint for different knee flexion angles using fluoroscopic and MR images [185].

 Numerical models have been validated against measurements to some extent. The *in situ* ligament forces and knee kinematics obtained from FEM were compared with the published experimental data [81]. MRIs from the sagittal plane were used to reconstruct the joint geometry. In this study, the femoral cartilage was assumed rigid and the tibial cartilage was deformable. The ligaments and meniscus were represented by equivalent spring elements [81]. In another study, a 2D FE model was constructed for a sagittal plane of a rabbit knee [186]. The tibia force predicted by the FE model was matched with the measured force. The study showed that advancement of calcified cartilage resulted in thinning of noncalcified cartilage and increased shear strains within its deepest layer. Small deformation was considered with absence of menisci [186].

 In order to validate a FE hip joint model for walking, stair ascending and descending, Anderson and coauthors measured contact pressures and areas using pressure-sensitive films [174]. CT images were processed using Amira (Mercury Computer Systems, Boston, MA, USA). TrueGrid was used for mesh generation, and NIKE3D (Livermore, CA, USA) was used for the finite element analysis. Cortical and trabecular bones were assumed as hypoelastic and isotropic. A custom code, BONEMAT [187], was used to calculate the elastic modulus of bones from measured data. The FE results were found to agree with the experimental measurements [174]. Similar validation procedure may be performed on the knee joint modeling.

 A 3D analytical model was used to simulate knee kinematics, where the menisci were not included. An elastic cartilage-cartilage contact was compared to a rigid femur-tibia contact [121]. The model was also validated against the laxity characteristics data from the literature. The kinematic data of knee specimens were used as the objective of an optimization procedure, and the ligament initial strains were altered to achieve the optimization, using a least-square solver [188].

 Yao and coauthors used MRI to validate a FE model of the medial compartment of an ACL-deficient knee subjected to anterior forces. The differences between FE predictions and data from imaging were noticeable for changes in curvature and distortions within anterior and posterior areas of the meniscus [189]. In a further study, they used the finite element model of the medial compartment of the ACL-deficient knee joint to reproduce experimental deformation and motion of the meniscus by optimization of the mechanical properties of cartilage, meniscus, and the attachments of meniscus. This study illustrated the importance of mechanical properties of meniscal attachments, such as the initial strains and elastic modulus of the horns, to predict the meniscal translation and deformation [30]. This is an example of using imaging technology to validate the modeling at the strain level.

## 6. Pathomechanical Modeling and Clinical Applications

A few of the previously mentioned studies considered some aspects of knee injuries [80, 86–88, 146, 158]. In fact, many FE models were developed to investigate the impacts of injuries and surgical treatments on joint mechanical functioning. In this section, we review some examples of computer knee models that were intended for clinical applications, such as studies on ligament injury and reconstruction, meniscectomy, cartilage injury, and knee replacement.

### 6.1. Ligament Injury and Reconstruction

Li and coauthors studied the effects of ACL injury on joint function under simulated muscle loads [190]. They modeled a partial ACL injury by reducing its stiffness. It was found that even with 75% reduction in the ACL stiffness, the tissue could still support about 58% of the load carried by the intact ACL [190]. Moglo and Shirazi-Adl investigated the load transmission in ACL-deficient joints under drawer (anterior posterior) loading. They reported a primary resistance to the drawer load for ACL in the range of 0–90 degrees of knee flexion angels [191]. Suggs and coauthors studied the effects of graft stiffness and its initial strains on ACL-reconstructed knee joints. Three different grafts with stiffness close to that of actual ACL were used in their study [108]. Peña and coauthors also studied the effect of graft stiffness and tensioning in ACL reconstruction [192]. They implemented a hyperelastic model of the ligaments instead of using nonlinear springs but did not consider cartilages and menisci in their modeling. Three different grafts, gracilis, patellar tendon, and quadrupled semitendinosus were considered in the simulations [192].

**Figure 6:**
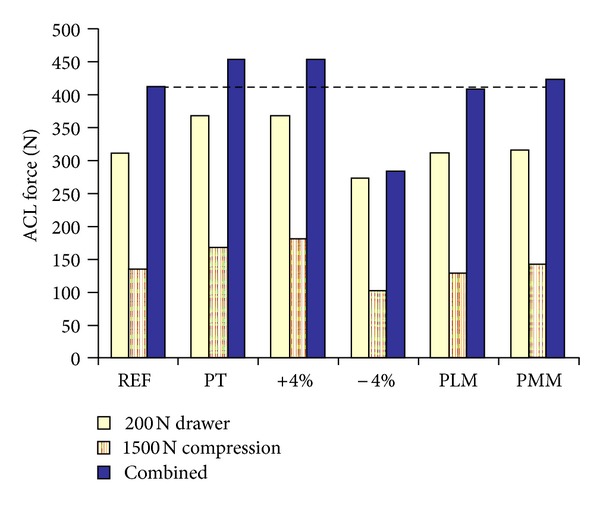
Predicted force in ACL under drawer load of 200 N and compression of 1500 N acting alone or combined. REF: reference case; PT: patellar tendon properties used for ACL, ±4%: 4% increase/decrease in ACL prestrain in each bundle; PLM/PMM: partial lateral/medial meniscectomy (reproduced from [148]; Elsevier license permission 2927920331018).

 Ramaniraka and coauthors studied the effects of PCL reconstruction on knee biomechanics. Ligaments were considered as hyperelastic. The healthy knee response was compared to that of three repaired knees: resected PCL, reconstructed single graft PCL, and reconstructed double graft PCL. The single graft reconstruction yielded better results compared to the other two cases [193]. In a further study, they evaluated the intra-articular and extra-articular procedures for ACL reconstruction using a knee model without cartilages and menisci [194]. Shirazi and Shirazi-Adl studied the effects of ACL reconstruction and partial meniscectomy under combined compression and drawer loads. They used a fibril-reinforced model for cartilages and menisci and spring elements for the ligaments. It was found that compressive preloads increased the ACL reaction forces in drawer loading (Figure 6). Moreover, partial meniscectomy combined with a slack ACL significantly changed the cartilage contact pressures [148].

### 6.2. Total and Partial Meniscectomy

Several FE studies have been performed to investigate the biomechanics of partial and total meniscectomy. Bendjaballah and coauthors studied the knee mechanics after total meniscectomy using a single-phase material model [80]. Kazemi and coauthors further considered the fluid pressurization in the cartilaginous tissues [87]. The impact of partial meniscectomy on fluid pressurization in cartilage was also investigated [88]. The model predicted significant increases in fluid pressure following partial meniscectomy. Peña and coauthors investigated the contact mechanics of meniscectomized knee joints and predicted almost double maximal shear stresses as compared to a healthy knee. They also suggested that a lateral meniscectomy was more risky than a medial meniscectomy [109, 142, 195]. Zielinska and Haut Donahue reported significant increases in contact pressures after meniscectomy using a linear elastic material model for cartilages and menisci [141]. Yang and coauthors studied the case of partial meniscectomy combined with frontal plane knee alignment. Increased contact stresses, with the highest increase in the lateral meniscectomy, was reported in their investigation. Cartilages were assumed as isotropic, and menisci were considered transversely isotropic in the study [111]. Wilson and coauthors developed an axisymmetric, poroelastic model of the knee to study potential cartilage damage after meniscectomy. They found that the maximum stresses and stress distribution in cartilage were altered after meniscectomy [105]. Netravali and coauthors studied the effect of partial meniscectomy on the meniscus strains during gait. They found that the increase in the abduction moment escalated the strains in the medial meniscal horns. Moreover, they suggested that the change in the external rotation after partial medial meniscectomy might not increase the chance of further medial meniscal degeneration [196].

### 6.3. Cartilage Injury and Degeneration, Osteoarthritis Models

The onset and progression of osteoarthritis (OA) are related to the mechanical environment of the tissue [197]. 3D models of the knee joint have potentially provided useful tools whereby the mechanics of cartilage degeneration can be better understood. Papaioannou and coauthors modeled focal surface injury of articular cartilage using a patient-specific FE model loaded at 30 degrees of knee flexion. They studied the size effects of osteochondral defect on contact pressures and reported a defect size of 10 mm as a threshold for clinical considerations of focal articular surface injury repair [136]. Shirazi and Shirazi-Adl investigated the effect of osteochondral defects on cartilage mechanical response [198]. In their model, depth-dependent properties of cartilage and fibers were considered, and the calcified cartilage was assumed as linear elastic and isotropic. Four different cases were considered: localized bone damage, cartilage-bone interface damage, bone overgrowth, and absence of collagen fibers in the deep zone. Significant change in joint contact mechanics was reported specially in the case of bone damage combined with cartilage split (which resulted in the absence of deep collagen fibers). Moreover, the results for cartilage-bone interface damage indicated increased chance of OA onset and progression [198]. A subject-specific study on the size effect of cartilage defect indicated a size threshold of 1.0 cm^2^ at which considerable change in cartilage stresses would occur around the defect rim [199].

 Peña and coauthors examined the effect of cartilage defects on stress concentration [200]. A hyperelastic transversely isotropic model was used for ligaments. The strain energy density function consisted of three parts: one represented the quasi-incompressibility of the tissue, one pertained to fibers in tension, and the third pertained to the matrix, which was assumed as Neo-Hookean. They reported that large cartilage defects produced high stress concentrations as compared to small defects [200]. Mononen and coauthors considered healthy, osteoarthritic, and repaired cartilages and developed a 2D knee joint model [139]. Different material models were compared for cartilage: isotropic poroelastic, transversely isotropic proelastic, and fiber-reinforced poroviscoelastic (FRPVE). In the FRPVE, the fiber direction and fluid content fraction were depth dependent, and a Neo-Hookean hyperelastic model was used for the nonfibrillar matrix. Their results demonstrated the important role of collagen fibers in controlling stress and strain distribution within cartilaginous tissues, which may be used in the design of artificial cartilages [139]. Later, they developed a 3D knee model in ABAQUS with four different split-line patterns for the cartilage. A random function in MATLAB (The Math Works Inc., Natick, MA, USA) was used for the model with random fibril orientations. The MRI reconstruction was performed using Mimics and SolidWorks. They concluded that a local cartilage degeneration in the medial femoral condyle could lead to alternation in mechanical response and a potential degeneration in the lateral condyle [112].

### 6.4. Knee Replacement

**Figure 7:**
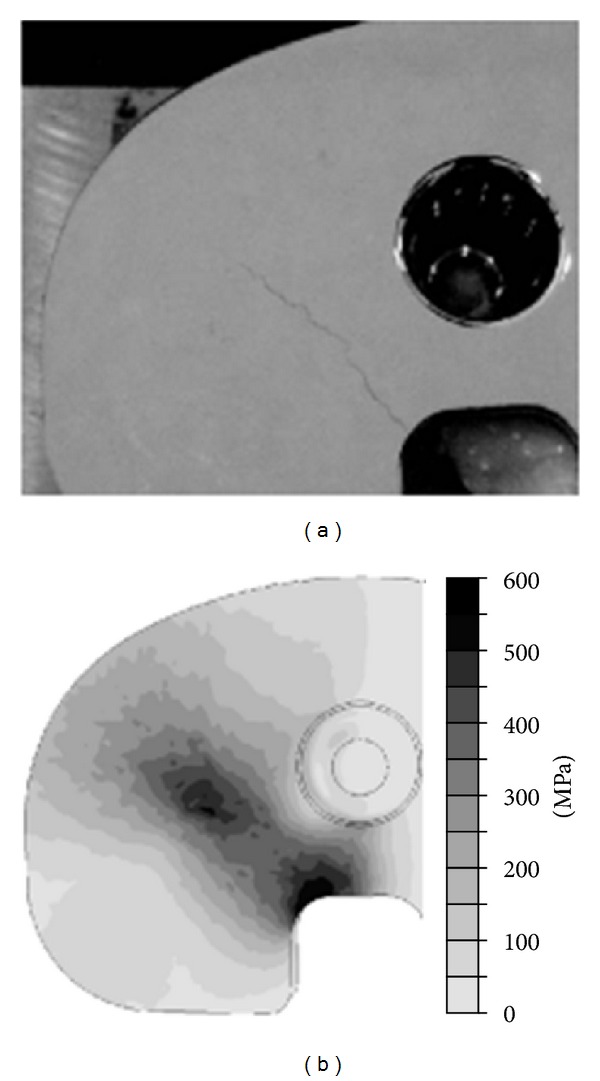
Experimental failure in the tibial tray of a knee implant due to fatigue loading (a) and von Mises stress from FE analysis (b) (reproduced from [149]; Elsevier license permission 2927920497991).

The mechanical performance of knee prostheses has been extensively investigated computationally. Godest and coauthors studied the kinematics and stress distribution of a total knee replacement (TKR) during a gait cycle using explicit FE code PAM-SAFE (Engineering Systems International Group, Rungis, France), which was reported to be computationally of low cost [201]. The gait cycle was simulated using a knee simulator composed of four springs. The femoral component of the prosthesis was assumed as rigid, and the insert was considered as an elastic-plastic material. The obtained kinematic results were in agreement with experimental data and were found to be insensitive to model parameters. The major sources of errors were reported from neglecting the mass of fixtures in the simulator, approximation of friction coefficient, and set-up errors such as relative position of the femoral component and the tibial insert [201]. Villa and coauthors studied the failure of a knee prosthesis during gait cycles, as well as fatigue [149]. They used Fuji Prescale films to determine contact areas and pressures. A standard ISO test with a small number of samples was used to validate the results for fatigue failure analysis. The obtained FE results were in agreement with the experimental measurements (Figure 7).

Danĕk and coauthors used FE modeling to determine contact in TKR based on geometries obtained from X-rays. According to the results, the outward condyles of the knee experienced higher pressure [202]. Sharma and coauthors computed the femoro-polyethylene contact pressure in total knee arthroplasty (TKA) using fluoroscopic images, CT scans, and Mechanical Desktop (Autodesk Inc, San Rafael, CA, USA). The contact pressures were calculated using forces obtained from kinematic modeling and contact areas obtained from computer-aided design of implants [203]. As a comparison, results were obtained for fixed bearing and mobile bearing TKAs. In both cases, the medial condyle experienced higher contact pressure. Furthermore, the contact pressure increased with knee flexion. The average lateral contact pressures for both TKAs were similar. However, the mobile bearing TKA experienced lower medial contact pressure compared to the fixed bearing TKA [203].

Au and coauthors examined the effects of material parameters and load conditions on stress distribution within the TKR [204]. They applied contact pressures on the tibia condyle using data from the literature and included ACL, PCL and MCL forces in the FE simulations that were performed using Pro/ENGINEER (PTC, Needham, MA, USA) and ANSYS. They suggested that in the design process of TKR, attention should be given to both material properties and loading conditions [204]. Bougherara and coauthors used ANSYS Workbench to analyze a TK implant made from CF/PA-12. Results showed that CF/PA-12 led to an improved load transfer mechanism and therefore reduced stress shielding, as compared to stainless steel [205].

Baldwin and coauthor validated a 3D dynamic model of the TKR against experimental data from a knee simulator. They used SCANIP (Simpleware, Exeter, UK) for MRI reconstruction, Isight (Simulia, Providence, RI, USA) for strain and stiffness optimization of ligaments, and ABAQUS/Explicit for FE simulations. In the modeling, ligaments were represented as 2D fiber-reinforced structures, and their mechanical properties were based on optimizations of laxity tests. FE results were reported to be in general agreement with experimental measurements [206].

A patient-specific implant design was proposed for unicompartmental knee replacement based on a neural network algorithm called Self-Organizing Map (SOM). The mechanical performance of this design was compared with conventional implant designs using MD Patran (MSC Software Corp., USA). Mimics, 3-Matic, and MATLAB software were used to reconstruct the 3D geometry of the samples from CT, MRI, and 3D laser scanner data. The femoral component was assumed as isotropic linear elastic, and the material properties of polyethylene bearings were modeled as nonlinear. A contact model based on the Hertz theory was used to validate the FE results. It was reported that the new mobile-bearing implant resulted in lower contact stresses in the tibiofemoral joint compared to the fixed-bearing implants. Moreover, lower stresses at the bone-implant interface were observed compared to other conventional implants [207]. A mobile bearing TKR was experimentally tested and numerically modeled using Patran and ABAQUS [208]. The polyethylene was considered as a nonlinear material for which the tangent elastic modulus was a fourth-order function of von Mises stress. Assessment on the effect of load conditions and flexion angle on the performance of the TKR demonstrated appropriate functioning under practical conditions. Large frictional loads at the mobile interface were reported as a major restriction on the TKR rotation [208].

### 6.5. Sports and Gait Modeling

The computational studies of the knee joint have mostly involved static loading conditions such as compressive forces and torques. More realistic loading conditions were indeed incorporated in some studies to simulate daily life activities.

**Figure 8:**
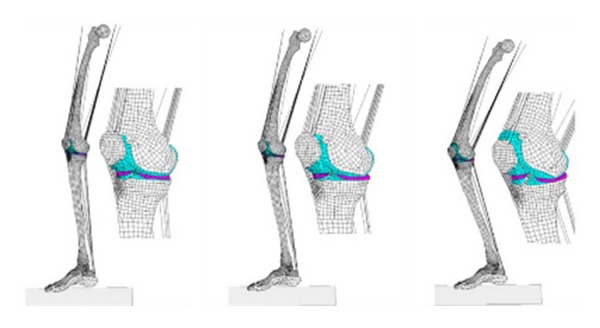
Finite element model of the knee joint included in the simulation of lower extremity. Three time points from the left to right are impact (*t* = 0), 10° of flexion (*t* = 0.02 s), and 30° of flexion (*t* = 0.074 s) (reproduced from [150]; Elsevier license permission 2927920646901).

 Penrose and coauthors constructed a 3D FE knee joint model to investigate the mechanics of the knee during stair descending, frontal car crash, and pedestrian impact. The model was suggested to be used for the design of prostheses and better understanding of biomechanics of injuries and locomotion [137]. A 3D leg model was built with the FE code RADIOSS (Mécalog SA, Antony, France) considering the entire lower extremity, including femur, tibia, major muscles, foot, and ankle complex (Figure 8). The kinematics and kinetics data were extracted from a gait analysis on a subject hopping on one leg. The measured forces and displacements were applied to the FE model of the knee as the boundary conditions. An elastic-plastic material law was used for cancellous and compact bones. Viscoelastic properties and synovial fluid were not modeled [150].

**Figure 9:**
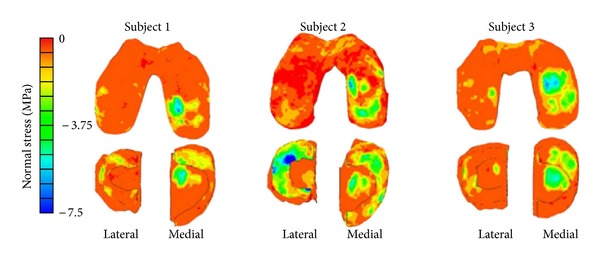
FE computed stresses for subjects with knee varus (1), normal subject (2), and with valgus (3) (reproduced from [151]; John Wiley and Sons license permission 2927360959287).

 ANSYS and LS-DYNA were used to develop an FE contact model of the knee joint in heel strike, single limb stance, and toe-off phases of a gait cycle. Results showed that the medial compartment experienced higher contact areas in comparison to its lateral counterpart. On the other hand, the lateral meniscus experienced steadier contact pressure compared to high variations in peak contact pressure in the medial meniscus. Furthermore, the peak contact pressure in the joint occurred at almost 45% of the gait cycle [209]. Yang and coauthors developed a 3D model of the knee joint with ABAQUS to investigate the effect of abnormal joint alignment and meniscectomy, during single stance phase of gait (see also Section 6.2, [111]). Ligaments were modeled as linear or nonlinear springs, and muscle forces were obtained using a muscle reduction method. These studies demonstrated the importance of using realistic loading to determine the knee joint mechanics [151, 210, 211]. For instance, while a simple compressive load to the joint produced almost equal contact forces in the medial and lateral compartments, a combined varus moment and compression (that occurs during gait) resulted in a much higher force in the medial compartment. Furthermore, as compared with a normal subject, a subject with varus alignment was more vulnerable to medial compartment OA, and a subject with valgus alignment was more vulnerable to lateral compartment OA (Figure 9). However, only a few subjects were used to obtain these results. The muscle forces used for the model input were not subject specific [151, 210, 211].

## 7. Miscellaneous Joint Models

While the focus of our paper is on the knee joint, some FE models of other human joints are briefly discussed here. This is because many features and principles are common in the computational modeling of different human joints. Some methodologies developed in other joint modeling may be applicable to the knee joint modeling and vice versa.

 A generic model of distal femur was produced from five cadaver knees, by reconstructing their CT images using AutoCAD (AutoDesk, Sausalito, CA, USA). The solid model was then prototyped using a MasterCAM's system (CNC Software, Tolland, CT, USA) controlled three-axis milling machine. Prosthesis design was mentioned as one of the potential applications of the generic geometry [212]. Ferguson and coauthors studied biomechanics of the acetabular labrum considering consolidation of cartilage [213]. A 2D plane strain model was reconstructed for the coronal plane of hip using MRI. Cartilage and labrum were considered as isotropic poroelastic materials. Results indicated important roles of the acetabular labrum in the mechanical function of the hip joint, for example, it improved the contact and stability of the joint [213]. Büchler and coauthors generated shoulder FE models of normal and osteoarthritic cadaveric joints [214]. For the osteoarthritic model, articular cartilage was assumed to be absent from the glenohumeral contact region. The humerus was modeled as rigid, and the scapula was considered as linear elastic but nonhomogeneous depending on the bone density. A custom-made software was used to determine the bone density from CT data. The muscles were considered as incompressible hyperelastic. The results indicated the importance of joint geometry on its contact mechanics [214]. Wawro and Fathi-Torbaghan developed an object-oriented FE program to study the motions of the knee joint. Femur, tibia, ligaments, and articular cartilage were modeled as elastic solids. The authors presented the framework of their long-term goal to develop a computer model of the knee joint based on object-oriented programming [215]. Han and coauthors used TrueGrid to generate a feline model of patellofemoral joint from laser scanning. Articular cartilage was considered as biphasic with deformation-dependent permeability. The geometric nonlinear option in ABAQUS was chosen for the FE analysis. They concluded that a small misalignment between patella and femur could lead to substantial changes in contact mechanics [216].

## 8. Discussion: Advances, Challenges, and Future Directions

A general review of the computational studies of the knee joint mechanics has been presented herein. Finite element methods have been generally accepted for the determination of the mechanical response of the knee in different loading and pathological conditions. The extensive applications of FE analyses have benefited and will continue to benefit from increased computational power. However, the computer power never seems to be sufficient for real-time simulation of the load response of a knee joint. Improved numerical procedures or brand-new techniques are still necessary for better and faster contact solutions. On the other hand, it will remain challenging to verify and validate a knee joint model. A few aspects of the computational joint mechanics will be discussed later.

### 8.1. Anatomically Accurate Geometry

Constructing an accurate geometry for the knee is an essential step for a successful modeling. Major progresses have been made in the geometry modeling. In the early studies, the knee joint was simply modeled with two pieces of articular cartilage, either axisymmetrical or plane-stress/strain. Meniscus was considered in some of these two dimensional models, for example, assuming one axisymmetric meniscus [164]. The actual knee, of course, is three dimensional with multiple gliding surfaces and interfaces. Patient-specific modeling presents realistic joint contact; however, it increases numerical difficulties and computational time by a few levels. Therefore, certain simplifications are often necessary in the anatomically accurate or patient-specific modeling. For example, in one of the pioneering studies, the femoral cartilage was modeled as rigid and menisci were modeled as springs [81].

 Accurate segmentation is still challenging. First, even with 3T MRIs, some tissue boundaries, for example, part of meniscus, are still difficult to identify from a computer screen. Secondly, even with advanced image processing software such as Mimics, enormous manual input is still needed. Thirdly, it often requires surface refinement before the geometry can be meshed with finite elements. We found limited tools and controls over the surface refinement. Artifacts and errors are difficult to determine with the currently available software.

 A good finite element mesh should preserve the reconstructed surface geometry, which is assumed to represent the original tissue geometry. This is particularly difficult for the meniscus meshing due to large thickness variation. Inaccurate surface approximation with element meshing will alter the contact in the joint and cause convergence difficulties. Future meshing software should provide better control and estimation of the surface errors produced during meshing.

### 8.2. Use of Constitutive Models

 Elastic models with compressible material properties were generally used for the cartilaginous tissues in the early patient-specific joint modeling. A Poisson's ratio close to half was used to approximate the incompressibility of the tissue at instantaneous compression. An effective Young's modulus, which was at least one order higher than the actual modulus obtained at equilibrium, must be used in order to match the predicted force with the measurement at fast knee compression. Although this effective modulus method might be used to determine certain stresses, it was not recommended for the deformation [32]. The incompressibility can never be approached within a compressible material model, not to mention the uncertainties encountered in determining the effective modulus. If only the solid phase is to be considered, an incompressible material model should be employed, as was done in a recent study [148].

 Another major progress in the course of model development was to incorporate the collagen fiber orientations in cartilage and menisci in the 3D model [198]. Since these tissues are anisotropic, it is important to determine the directions of the stresses and strains using the fiber orientation as a frame of reference: a smaller tensile stress in the direction perpendicular to the fibers may be more risky to tissue integrity than a larger tensile stress in the fiber direction. The von Mises stress is not a useful measure in the mechanics of anisotropic materials, because it does not discriminate the stress directions. In spite of this, use of the von Mises stress is still justified in the case of isotropic material modeling, when simplicity is desired.

 It is certainly challenging to incorporate fluid pressurization in the cartilaginous tissues into the 3D modeling. It is worth our efforts to work on it though. The fluid pressurization clearly plays important roles in the mechanical functioning of the joint. We may not understand OA onset and progression if the fluid mechanism in the tissues is overlooked. Nonlinear fibril reinforcement, however, must be incorporated in the material model in the meantime or the fluid pressure predicted for fast compression will be one order too low in magnitude. This is because the interplay of fibril reinforcement and fluid pressurization determines the load response of the tissue [33]. A material model that considers the fluid phase but no fibril reinforcement has been proven unable to describe great fluid pressurization in articular cartilage [45–48]. If fibril reinforcement were not modeled, the effective modulus method would have to be used in conjunction with the inclusion of fluid pressure, in order to match the load response measured for fast knee compression. The load response predicted for subsequent equilibrium state, however, did not match the measurement because the modulus used was greater than the actual modulus. In other words, such a model would not be able to predict both short-term and long-term load responses. This can be easily understood using creep as an example. When the body force is quickly applied to the knee, a great fluid pressure will be produced in articular cartilage, which cannot be described by a model with no fibril reinforcement. Using a large effective modulus in the FEA, however, one can still match the small short-term displacement associated with great fluid pressure. The displacement will later increase substantially when fluid pressure essentially disappears at equilibrium. This displacement obviously can only be described by the actual modulus, which is obtained by tissue testing at equilibrium. The use of effective modulus will predict a smaller displacement than the one observed at equilibrium.

 The computation of fluid pressurization in the joint is extremely time consuming. One simulation often takes days even weeks of computer time. An obvious reason is the necessity to calculate the results for hundreds or thousands of time increments, because every unknown is a function of time. The main obstacle, however, is the convergence difficulties associated with the contact problem of porous media, which is even worse with the multiple contacts in the knee joint. As the menisci are in double contacts with cartilages—sandwiched by the femoral and tibial cartilages, the contact convergence is particularly slow. Wilson and coauthors used a thin flexible membrane between the menisci and cartilage surfaces to avoid numerical difficulties in an axisymmetric model of the knee. Mononen and coauthors considered the fluid flow in cartilages in a 3D model of the knee undergoing large deformation, with the menisci modeled as a single-phase transversely isotropic material [112]. In our research group, we initially examined the 3D knee modeling with small deformation problems following the implementation of the fluid flow and collagen orientation in cartilages and menisci [86–88, 138, 217].

 The accuracy of constitutive models is ultimately determined by the material properties of the tissue. Several studies have indicated the importance of reliable material properties in the modeling and the sensitivity of results to the properties [7, 23, 30, 32, 65, 67, 85, 204]. Clearly, it is still challenging to adequately measure and quantify the material properties. For instance, the inhomogeneous, anisotropic, and time-dependent nature of articular cartilages, menisci, and ligaments requires several material parameters at the joint, tissue, and cell levels. These aspects must be considered in order to establish computational modeling as a robust tool for predicting the load response of the knee joint.

### 8.3. Physiological Loadings and Contact Conditions

No mathematical modeling is possible without simplifications associated with assumptions, even with future advances in computational hardware, software, and numerical techniques. A few common simplifications are often made in the 3D knee joint modeling, such as the use of static compressive loadings, passive muscle forces only, or omission of muscles and tendons. In addition to the simplifications associated with the geometry and constitutive laws discussed previously, another major simplification is the consideration of one contact state corresponding to a single stance of a gait cycle, other than a dynamic contact in which the contact region moves with knee flexion. Current gait modeling is typically limited to the kinematics of the knee or the total forces and moments in the joint without much concerns over the contact and fluid pressures in the joint.

 For a quasistatic problem, where the fluid pressurization is considered but the inertia is neglected, the pressure gradients in the tissues will critically influence the rate of convergence [87]. A fast loading will result in slow numerical convergence. Even with the simplest knee compression in the femoral tibial direction with no rotation or flexion, a compression applied within a realistic time, which is normally less than one second, will cause very slow convergence [87, 88]. In a dynamic contact, for example, with knee flexion as a function of time, part of the surfaces that are currently in contact may separate milliseconds later. This will change the boundary conditions of fluid pressure, since a zero pressure condition must be imposed on the free articulating surface, which is not in contact with the mating surface. Such changes may also slow down the numerical convergence, not to mention the technical difficulties in applying the free surface boundary conditions using a numerical procedure.

 There are at least two major causes with the slow computation of the fluid pressure modeling in the patient-specific knee joint. First, the response as a function of time requires hundreds and thousands of time increments in the time discretization, while no time variable is involved in a static analysis with an elastic modeling. Second, when ABAQUS (version 6.10 or older) is used, the contact convergence with 20-node hexahedral elements is very slow, which is indicated in the manual. However, this type of elements is needed for better pressure distribution. We are not sure whether the same problem exists with other commercial FE packages.

 The numerical convergence for a problem of walking can be incredibly challenging, if fluid and contact pressures are to be determined. A typical remedy is still to avoid modeling fluid pressure and adopt an incompressible elastic constitutive behavior for the cartilaginous tissues [93, 148]. The fluid pressure dissipation during a gait cycle should be negligible, because the loading cycle is in the order of a second [93], while creep takes thousands of seconds to complete. However, the material incompressibility must be explicitly formulated, other than using an effective modulus and a Poisson's ratio close to half to approximate the incompressibility [32].

 With all the difficulties discussed so far, it would be hard to imagine the endeavors required to simulate cyclical loadings when fluid pressure is considered. We have performed some preliminary investigations to simulate a person standing on a vibration plate, so no knee flexion needs to be considered. This test condition made it possible to run the computations (results not published yet). Frequency-dependent load response has been investigated using cartilage explants [218–220]; it would be interesting to understand the relevant behavior with the knee joint.

### 8.4. Future Directions

 Although major progresses have been made in the last two decades, much work remains to be done in the computational knee joint mechanics, for example, streaming potentials in the knee have not been modeled so far. However, we will not attempt to discuss detailed research topics here, because they are related to variety research interests and goals of individual research groups. Instead, we would like to discuss some general issues with the model development.

#### 8.4.1. Model Verifications

Model verification has probably not been paid as much attention as model validation. Verifications are particularly important when limited experimental data are available for the validation of the joint modeling. If the material model is valid, geometry reconstruction is right, and every aspect of the numerical procedures has been proven to be correct, then the joint model is likely valid even without experimental validation. A knee joint model should be acceptable, after complete verifications have been done. A few additional aspects are deliberated here, besides the validation of geometry reconstruction discussed previously.

 Verification of the contact approach for the particular problem is very important because mechanical contact is a key issue over the numerical convergence. The verification can be performed with simpler contact geometry and loading conditions, with which the numerical results can be more easily understood. Contact definitions and parameters should all be tested before they are used in an anatomically accurate joint model. The contact approach can also be validated using a simple indentation testing, if the material model for the specimen has been validated.

 The analysis procedure must also be tested for the desired bioengineering application. Since the majority of commercial FE packages were originally developed for structural analysis or traditional engineering applications, the solution procedure may not be optimized for any biomechanical analysis. A few parameters are usually provided in a commercial software for the customer control of the analysis. It must be noted that the default values set by the software may not be the best for the contact mechanics of the knee, although they may be the best for the structural analysis of a robot. For example, the Soil Consolidation procedure from ABAQUS has been widely used to simulate the quasistatic response of articular cartilage. When using this analysis procedure, we should first note that the elastic material model in ABAQUS does not include the feature of fibril reinforcement observed in cartilage. Therefore, a user-defined stress-strain relationship is recommended for the tissue matrix. In addition, the definition of permeability in ABAQUS complies with the practice in civil engineering, which must be adapted to the usage in biomechanics. Finally, the control over convergence and accuracy is quite tricky. One must choose the right combination of maximum allowable time and pore pressure increments for each step. Otherwise, the convergence can be very slow or may never be achieved.

 Recently, an open source nonlinear, implicit FE program called FEBio has been introduced that is designed and tailored for biomechanical simulations (http://mrl.sci.utah.edu/software/febio). The software currently supports computational solid biomechanics and as an open source package has the potential to expand by the users for particular problems. The software is based on C++ computer language, supports parallel processing, and has its own pre- and postprocessors called PREVIEW and POSTVIEW, respectively [221]. A feature of the program is to allow fluid flow across the contact interface, although this feature becomes available in the two new versions of ABAQUS (v6.11 & v6.12).

 Inappropriate finite element meshing may produce incorrect results. It is known that the numerical solution must converge with the mesh refinement. However, the issue with knee joint meshing is over the distorted elements generated by automatic meshing to fit the complex tissue geometry. These elements should not be further refined, but be manually adjusted to speed up numerical convergence. The selection of tetrahedral versus hexahedral elements for mesh generation may also affect the accuracy of the results. While the discretization of a complex domain is much easier using tetrahedral or a combination of tetrahedral and hexahedral elements, such a mesh might not be suitable for some simulations. For instance, if porous elements in ABAQUS are used to model the fluid pressure, quadratic hexahedral elements yield better results. Moreover, the contact convergence is generally slower when triangular/tetrahedral elements are used as compared to quadrilateral/hexahedral elements.

 The verification of the joint model should also be performed with various loadings corresponding to the mechanical functions of the joint.

#### 8.4.2. Model Validations

Model validation is probably still weak in the computational modeling of the knee joint. Many validations in the past were limited to match partial measurement, for example, the total force in the joint, by choosing model parameters, such as contact conditions, geometric constraints, and material properties. Such matches may indicate certain procedures in the modeling have been done correctly but do not really demonstrate sufficient proof for the model validity. There are two issues with this type of validations. First, the material properties may not be in the right range, or the parameters are not physically correct at all. Secondly, only partial measurement is matched, which is often possible by adjusting multiple parameters at a reasonable range, even the modeling is not completely right. The real question is whether the model can be used to match multiple measurements simultaneously, for example, the use of one set of parameters predicts the total force, as well as maximum stresses and strains at various loadings. Since multiple measurements are currently difficult, this type of validation should be complemented with other types of validations. *In vivo* MRI measurement of soft tissue deformation may be promising in this regard.

 The validation of joint modeling should be focused on the choice of validated material models (constitutive laws), before new reliable techniques are further available for precise measurement of multiple mechanical parameters of the knee. Using articular cartilage as an example, a valid material model should be able to describe at least the load responses of both unconfined compression and tensile testing at different loadings. The unconfined compression testing is used to demonstrate the mechanism of fluid pressurization in the load support of the tissue, whereas the tensile testing is used to observe the intrinsic viscoelastic properties of the tissue. Successful simulation of the two types of testing indicates some capability of the proposed constitutive law in describing the two key mechanical mechanisms of articular cartilage. A confined compression testing can be further considered because it reveals the compressive properties of the tissue independent of the tensile properties. Mathematically speaking, the multiple material properties of a model can only be determined by multiple testing. Indentation testing, on the other hand, may not be as effective as the other types of testing for the validation of a material law, because the contact modeling itself for the indenter and specimen requires validation as well. One may also suggest 2D tensile tests for the validation of a material model [222].

 The validation of a material model must be performed for various loading magnitudes and loading rates, since tissues like cartilage and ligaments are well known for their nonlinear behavior and strain rate sensitivity [31–33]. It is noteworthy that multistep ramp loading and relaxation or creep tests well demonstrate the nonlinearity at both loading phase and equilibrium. These multistep tests may be best used to validate the constitutive law of a viscoleastic material [45, 47].

### 8.5. Concluding Remarks

A good knee joint model should be at least extensively verified. The validation may be focused on the constitutive laws of the tissues, because tissue testing is more developed and effective than whole joint testing. Joint measurements should also be performed to validate the numerical solutions whenever possible. Recent works in whole joint testing may provide data to validate the computational models in terms of kinematics/kinetics. For instance, joint simulator robots are capable of reproducing joint kinetic data when *in vivo* kinematics data are provided as inputs, or they can generate kinematic data emulating biological motions if kinetics data are given [223–225]. However, one should be aware of the limitation of all validations: limited experimental data are not sufficient for the validation of the joint model. Extensive verification of the model is still necessary, even the numerical results match the data well. Naturally, the model verification should be done prior to the validation of the joint model.

 A single run of FEA on the knee takes days or weeks, if the time-dependent response is sought, for example, the determination of the fluid pressurization in articular cartilages and menisci. In particular, creep takes a much longer time to reach equilibrium than stress relaxation [88]. If a valid material model is used, the creep response can be qualitatively derived from the relaxation testing. Therefore, we suggest to examine the stress relaxation of the knee first; even creep is considered as a more realistic loading. The simulation of creep is necessary only when quantitative results for creep are needed. Another major factor that slows down the computation is the use of actual fast loading, which is necessary for the prediction of realistic load response that is highly compression rate dependent. On the other hand, one may use a slightly slower loading to obtain much quicker convergence and yet acceptable results, since the rate dependence is asymptotic [226]. This method is effective only when the load response is close to the asymptote, which is the instantaneous response.

 The choice of a knee model depends on the research questions to be answered. A rigid-body model may serve the purpose of gait analysis of healthy knees. However, a FE model is required to determine the contact pressures in the knee in order to understand an abnormal gait. An FE knee model with a single-phase incompressible material law may be sufficient for the analysis of gait cycles but will not provide any information on the nutrient transport in articular cartilage that is performed by fluid flow in the tissue. A fibril-reinforced poromechanical model may also help understand the load share between the solid matrix and fluid pressurization, as well as the stress in the collagen network.

 The choice between implicit and explicit solution techniques is another factor that should be considered for numerical simulations. Implicit methods are preferred for static and quasistatic problems, while explicit methods are usually used for impact and fast loading problems [150, 201, 206]. Since explicit methods are conditionally stable, the criteria must be set carefully to ensure numerical stability and convergence [150]. Implicit methods are usually unconditionally stable, but as discussed earlier, the selection of convergence criteria is a critical aspect to ensure the numerical accuracy. It is noteworthy that most of the knee joint models cited in this review employ implicit methods. Another challenge, for a computationally demanding simulation, is the efficiency of parallel processing: explicit simulations are normally more efficient when the number of CPUs increases as compared to the implicit methods. In addition, when a commercial FE package is used, the number of licenses is a practical restriction on the number of CPUs recruited for a parallel computing. Finally, GPU supercomputing may be a future solution for real-time simulation of the knee joint mechanical response, which is beyond the scope of this paper.

## Figures and Tables

**Table 1:** Classification of constitutive models of knee tissues used in the literature for the computational modeling of the knee joint.

Tissue	Material model							
	Single-phase (solid phases only)							Poromechanical
	Rigid	Spring elements	Linear elastic		Hyperelastic	Viscoelastic	Fiber-reinforced	Fiber-reinforced
			Isotropic	Transversely isotropic				
Bones	[80, 81, 84–88, 97, 107–111, 113–116, 121, 137, 143, 148, 150, 158–160, 190–195, 199–201, 206, 213]		[32, 105, 110, 136, 138, 144–146, 150, 152, 157, 198, 209, 215]					

Articular cartilages	Rigid femoral cartilage; deformable tibial cartilage [81]		[18, 80, 81, 84, 85, 97, 105, 108–111, 114–116, 121, 136, 137, 142, 143, 145, 146, 150, 152, 157–160, 190, 191, 193–195, 199, 200, 209]	[105]	[113, 174, 214]		[113, 148, 198]	[32, 86–88, 112, 138, 139]

Menisci		[81, 107, 108, 143, 190]	[18, 84, 105, 109, 142, 150, 157, 193–195, 199, 200, 209]	[85, 97, 110–112, 136, 160]	[113]		[80, 113–116, 148, 158, 159, 191, 198]	[32, 86–88, 138, 139]

Ligaments		[80, 81, 97, 107, 108, 110, 111, 113–116, 143, 148, 150, 158, 159, 190, 191, 206, 210, 211]	[215]		[18, 84, 85, 109, 142, 192–195, 199, 209]	[86–88]	[206]	
